# Medical ultrasound image speckle reduction and resolution enhancement using texture compensated multi-resolution convolution neural network

**DOI:** 10.3389/fphys.2022.961571

**Published:** 2022-11-14

**Authors:** Muhammad Moinuddin, Shujaat Khan, Abdulrahman U. Alsaggaf, Mohammed Jamal Abdulaal, Ubaid M. Al-Saggaf, Jong Chul Ye

**Affiliations:** ^1^ Center of Excellence in Intelligent Engineering Systems, King Abdulaziz University, Jeddah, Saudi Arabia; ^2^ Department of Electrical and Computer Engineering, King Abdulaziz University, Jeddah, Saudi Arabia; ^3^ Department of Bio and Brain Engineering, KAIST, Daejeon, South Korea; ^4^ Kim Jaechul Graduate School of AI, KAIST, Daejeon, South Korea

**Keywords:** ultrasound imaging, deep learning, resolution enhancement, ultrasound image enhancement, convolution neural network

## Abstract

Ultrasound (US) imaging is a mature technology that has widespread applications especially in the healthcare sector. Despite its widespread use and popularity, it has an inherent disadvantage that ultrasound images are prone to speckle and other kinds of noise. The image quality in the low-cost ultrasound imaging systems is degraded due to the presence of such noise and low resolution of such ultrasound systems. Herein, we propose a method for image enhancement where, the overall quality of the US images is improved by simultaneous enhancement of US image resolution and noise suppression. To avoid over-smoothing and preserving structural/texture information, we devise texture compensation in our proposed method to retain the useful anatomical features. Moreover, we also utilize US image formation physics knowledge to generate augmentation datasets which can improve the training of our proposed method. Our experimental results showcase the performance of the proposed network as well as the effectiveness of the utilization of US physics knowledge to generate augmentation datasets.

## 1 Introduction

Ultrasound imaging is one of the most extensively utilized medical imaging procedures. Among the various benefits of the US imaging, the most prominent ones are its radiation-free and non-invasive nature. It is considered to be a versatile tool for scanning almost all body tissues with its applications in cardiology, gynecology, obstetrics, vascular imaging, and abdominal imaging among others. Despite its many benefits, ultrasound imaging technology has a significant image quality disadvantage when compared to other medical imaging modalities like x-rays, magnetic resonance imaging, and computed tomography (Sanches et al., 2012). Ultrasound images are typically noisy to the observer, and good correlation of anatomy/disease to the acquired ultrasound images necessitates extensive and long-term training.

Commercially available US systems/scanners generate images using the echo imaging principle. Pulses of acoustic waves with frequencies ranging from 1 MHz to 20 MHz are transmitted into the target tissues by means of a handheld transducer/probe which acts as a transceiver. The transmitted acoustic waves then interact with the tissues and some of the transmitted energy is reflected back and detected by the US transducers. Some key advantages of US imaging systems over other radiography/medical imaging methods can be:

• Low-cost systems as compared to computed tomography and magnetic resonance imaging
• Can work in real-time
• Noninvasive and radiation-free
• Compact and portable

Key limitations of the US imaging systems include their limited penetration into the tissue and the requisite skill of the sonographer or physician is required to have useful insights from the examination.

The presence of speckle noise in US images makes them appear noisy, which can mask pathological changes in the body and lead to diagnostic mistakes. As a result, since the early days of ultrasound imaging, the problem of speckle reduction in ultrasound images has been a focus of research for many academic and industrial research organizations, and it is predicted to continue so, given its impact on this technology (Loizou and Pattichis, 2015). Blood capillaries and cells in the extracellular space operate as scatterers, while tissue interfaces and large blood arteries act as speckular reflectors (Burckhardt, 1978; Wagner et al., 1983). As a result, image deterioration occurs in ultrasound imaging.

Early approaches to address the problem of noise reduction in US images include Anisotropic diffusion methods (Yu and Acton, 2002; Sun et al., 2004; Aja-Fernández and Alberola-López, 2006; Liu et al., 2011), Probabilistic Patch-Based (PPB) filtration (Deledalle et al., 2009), bilateral filter (Balocco et al., 2010; Tang et al., 2010), and the non-local means (NLM) (Zhan et al., 2014). Most of such contemporary techniques lack in performance due to sensitivity to signal dynamic-range and noise level, selection of patches, selection of algorithm parameters, computational complexity of the algorithm, etc. Among the NLM filtering techniques, a few NLM filtering approaches designed primarily for general image processing applications use low-rank information such as noise reduction in images (Gu et al., 2014), multispectral image denoising (Xie et al., 2016), and image deblurring (Dong et al., 2015). A major limitation of conventional methods is that they are designed to remove specific noise only, and doesn’t not improve the overall image quality e.g., resolution. Therefore, such methods might not be suitable for variable resolution images and may perform inferior to reduce speckle noise in US images. Furthermore, as there is no specific method to find candidate patches of the speckle patterns present in US images, global filtration may introduce blurring. In this regard, a low-rank non-local filtering-based speckle removal system is presented (Zhu et al., 2017; Li et al., 2018) which utilizes a guidance image that can assist the selection of candidate patches for non-local filtering, however, it is an iterative optimization-based method which is computationally very expensive.

Many researchers have examined deep learning approaches for US image enhancement challenge as a result of recent breakthroughs in AI, and have claimed that deep learning-based strategies can improve real-time image interpretation to enable quick and efficient decisions (Shokoohi et al., 2019). The recent finding of a close relationship between deep neural networks and Hankel matrix decomposition (Ye et al., 2018) prompted a major effort related to efficient ultrasound imaging utilising deep learning. The research revealed that the receiver-transmit (Rx-Xmit) and receiver-scan-line (Rx-SL) domains have considerable redundancy, resulting in a low-rank Hankel matrix (Jin et al., 2016). Convolutional Neural Networks (CNN) are preferred to be applied to the Rx-Xmit domains to utilise the redundancy in the RF domain (Yoon et al., 2018). In a similar direction, the work in (Khan et al., 2020a) presents the first deep learning-based adaptive and compressive beamformer for high-resolution ultrasound imaging. Recently, the studies in (Huh et al., 2020; Khan et al., 2021d) described some theoretically justified unsupervised algorithms for ultrasound image enhancement. The work in (Dietrichson et al., 2018) puts forward a deep convolutional generative adversarial network-based real-time ultrasound speckle noise reduction approach where three alternative sized generator networks were tested to analyze the performance-run-time trade-off. A fully convolutional neural network-based ultrasound B-mode imaging approach for producing speckle-reduced B-mode US images that uses a log-domain normalization-independent loss function in training is presented in (Hyun et al., 2019).

In contemporary methods, denoising often causes blurring in the final output subsequently reducing the resolution which is already limited due to the fundamental limitations of US physics. A variety of strategies have been proposed to address this problem, ranging from adaptive beamforming (Khan et al., 2020a) for side-lobe suppression to deconvolution ultrasonic methods (Duan et al., 2016). Because resolution loss is caused by a variety of reasons such as restricted bandwidth, speckle noise, and the number of channels, as a result, adaptive and tunable deep learning can be applied in this case (Khan et al., 2021b). Furthermore, when imaging physics is fully or partially known, such as when a blurring kernel or point-spread function (PSF) is understood, an explainable AI can be created to increase quality characteristics such as resolution (Khan et al., 2021d). Bind deconvolution is a method for predicting PSF and high-resolution images (also known as tissue reflectivity function (TRF)) from low-resolution images. Model-based deconvolution methods are dependent on a number of assumptions and do not operate in the presence of noise. A deep learning-based deconvolution approach has recently been proposed that does not require PSF estimate and can deconvolve RF data directly (Khan et al., 2020a). Similarly, a theoretically justifiable deep learning aided ultrasound image enhancement system is presented in (Khan et al., 2021d), where artificial intelligence-based speckle denoising is performed on phantom dataset for delay-and-sum (DAS) conventional beamforming images. The abality of learning complex patterns in a data-driven fashion motivated various researcher to exploit it for US image enhancement tasks, a short summary of research conducted on deep learning-based US imaging systems is presented in Table 1.

**TABLE 1:** A short summary of research conducted on deep learning-based US imaging.

Problem	Technique	References
Speckle Noise Reduction and Image Quality Enhancement	Multi-Resolution CNN	Vedula et al. (2017)
Speed of Sound Calibration	Deep CNN	Salehi et al. (2017)
B-Mode US Image Reconstruction	Deep CNN	Yoon et al. (2018)
US Artefact Reduction	CNN	Vedula et al. (2018)
US Super-Resolution	SRGAN	Choi et al. (2018)
Speckle Noise Reduction	ResNet-based GAN	Mishra et al. (2018)
Speed of Sound Estimation	Deep CNN	Anas et al. (2019)
Speckle Noise Reduction and Beamforming	Deep CNN	Hyun et al. (2019)
B-Mode US Tongue Feature Extraction	Denoising AutoEncoder	Li et al. (2019)
Speckle Noise Reduction	Denoising Autoencoder	Karaoğlu et al. (2020)
Phase Aberration Correction	Deep Auto-Encoder	Jeon and Kim. (2020)
Phase Aberrator Profile Estimation	Deep CNN	Sharifzadeh et al. (2020)
Super Resolution of B-Mode US Images	Deep CNN	Temiz and Bilge. (2020)
US Image Artefact Removal	Optimal Transport-Driven Unsupervised Deep CNN	Huh et al. (2020)
Speckle Noise Reduction	Mixed attention mechanism-based residual UNet	Lan and Zhang. (2020)
Speckle Noise Reduction and Contrast Enhancement	MimickNet (based on GAN)	Huang et al. (2020)
B-Line Assessment in Point-of-Care US	Deep CNN	Baloescu et al. (2020)
Assisted Diagnosis of Lung Disease in Point-of-Care US	Deep neural network	Roy et al. (2020)
Cardiac Point-of-Care US Image Quality Enhancement	Constrained CycleGAN	Jafari et al. (2020)
US Image Quality Enhancement	Unsupervised Deep Deconvolution Model	Khan et al. (2020b)
Estimation of Ultrasound Echogenicity Map from B-Mode Images	UNet Convolutional Neural Network	Shen and Yang (2020)
Speckle Noise Reduction and Beamforming	Deep CNN	Khan et al. (2021b)
Speckle Noise Reduction and Image Quality Enhancement	Deep CNN	Ma et al. (2021)
Contrast and Resolution Enhancement in POCUS	Self-consistent CycleGAN	Khan et al. (2021a)
US Artefact Removal	Unsupervised Optimal Transport CycleGAN	Khan et al. (2021d)
Accelerated Echocardiography with Artefact and Speckle Reduction	Unsupervised CycleGAN	Khan et al. (2021c)
Phase Aberration Correction	Self Supervised Learning	Khan et al. (2022)
Enhanced 3D ultrasound	Switchable CycleGAN	Huh et al. (2023)
Resolution and contrast enhancement	OT-drived CycleGAN	Huh et al. (2022)

Motivated by the recent trends in deep learning-based US image enhancement, in this work, we propose a deep convolution neural network (CNN) based US image enhancement method where the task of noise suppression and resolution enhancement are carried out simultaneously in a single network. For the task of noise reduction, we use a UNET-like architecture which is followed by a small convolution network where multi-scale features are incorporated to enhance the texture cues in the noise-free image. Since US image datasets are generally small datasets with only a limited number of real-world US images, the performance of deep learning techniques might degrade as such methods require large volumes of data. In order to improve the performance of the proposed method, we also devised an augmentation dataset which is designed by incorporating US image formation physics information.

The rest of the paper is organized as; section 2 outlines the proposed method, the experimental setup, performance evaluation parameters, and results are presented in section 3 followed by conclusion in section 4.

## 2 Materials and methods

In this section, we present our proposed deep CNN-based US image enhancement method. The contributions made in this work are two-folds; 1) we present a novel deep CNN based architecture for US image enhancement, 2) we also propose to incorporate US image formation physics into the training dataset to achieve better overall image quality. First, we need a denoising model for US image speckle noise reduction. Second, the output of the denoising model will be fed to our proposed resolution enhancement model; however, since resolution enhancement is susceptible to noise amplification, we suggest that the noise be reduced prior to the resolution enhancement task. Finally, we can combine both models to design an end-to-end deep CNN model that can remove speckle noise and improve resolution. The authors believe that the proposed approach is one-of-a-kind in two ways; first, the presence of densely connected convolution blocks using skip pathways enable efficient and accurate training of the proposed desnly-connected UNET-type CNN as suggested by (Huang et al., 2017), and secondly, the use of a dedicated resolution enhancement network after the densly connected UNET-like CNN.

### 2.1 US image formation physics informed data augmentation

**FIGURE 1:**
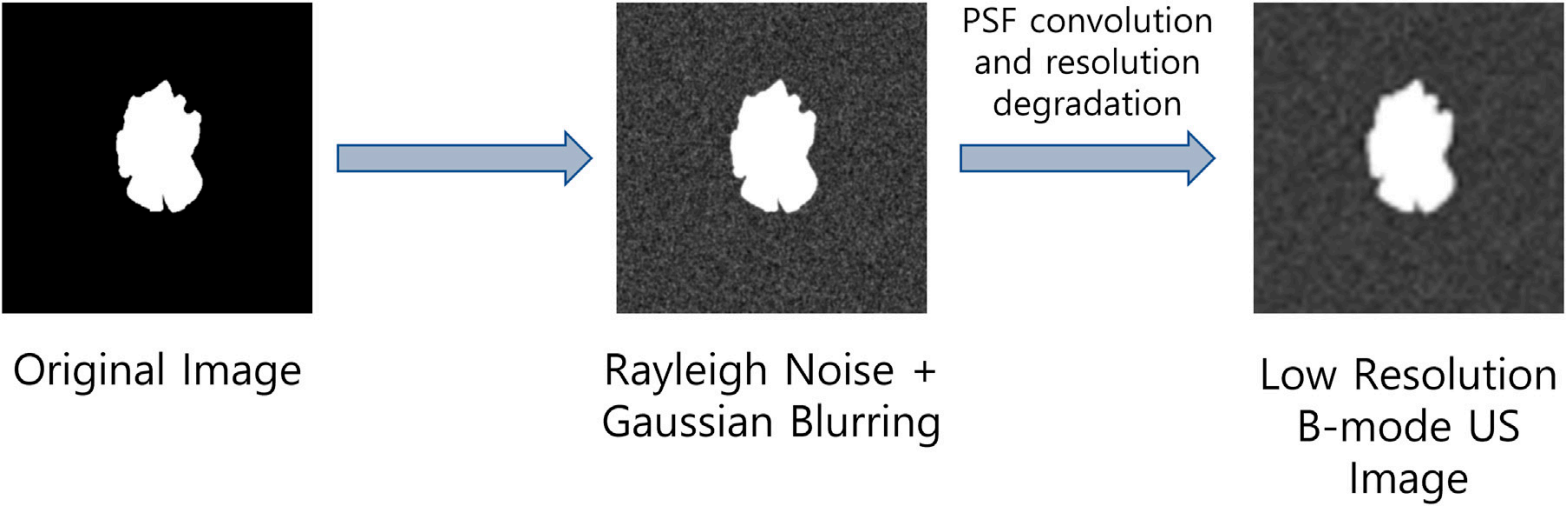
Ultrasound image formation physics informed dataset generation.

Since real-world US image datasets are usually small, hence training deep learning models with such low volumes of data may result in performance degradation as well as it can cause poor generalization of the deep learning models due to overfitting. To address these problems, we propose to perform data augmentation by introducing the US image formation physics information into the salient object detection (SOD) dataset presented in (Xia et al., 2017). To obtain low-resolution and noisy images, we corrupted the images from the SOD dataset with different Rayleigh noise profiles and apply Gaussian blurring with different variance profiles to obtain reflectivity amplitude. Finally, we define a PSF and convolve it with the reflectivity amplitude to obtain B-mode ultrasound-like images with speckle noises as suggested in (Shen and Yang, 2020). The US image formation physics informed dataset generation is highlighted in Figure 1.

### 2.2 Proposed US image enhancement method

**FIGURE 2:**
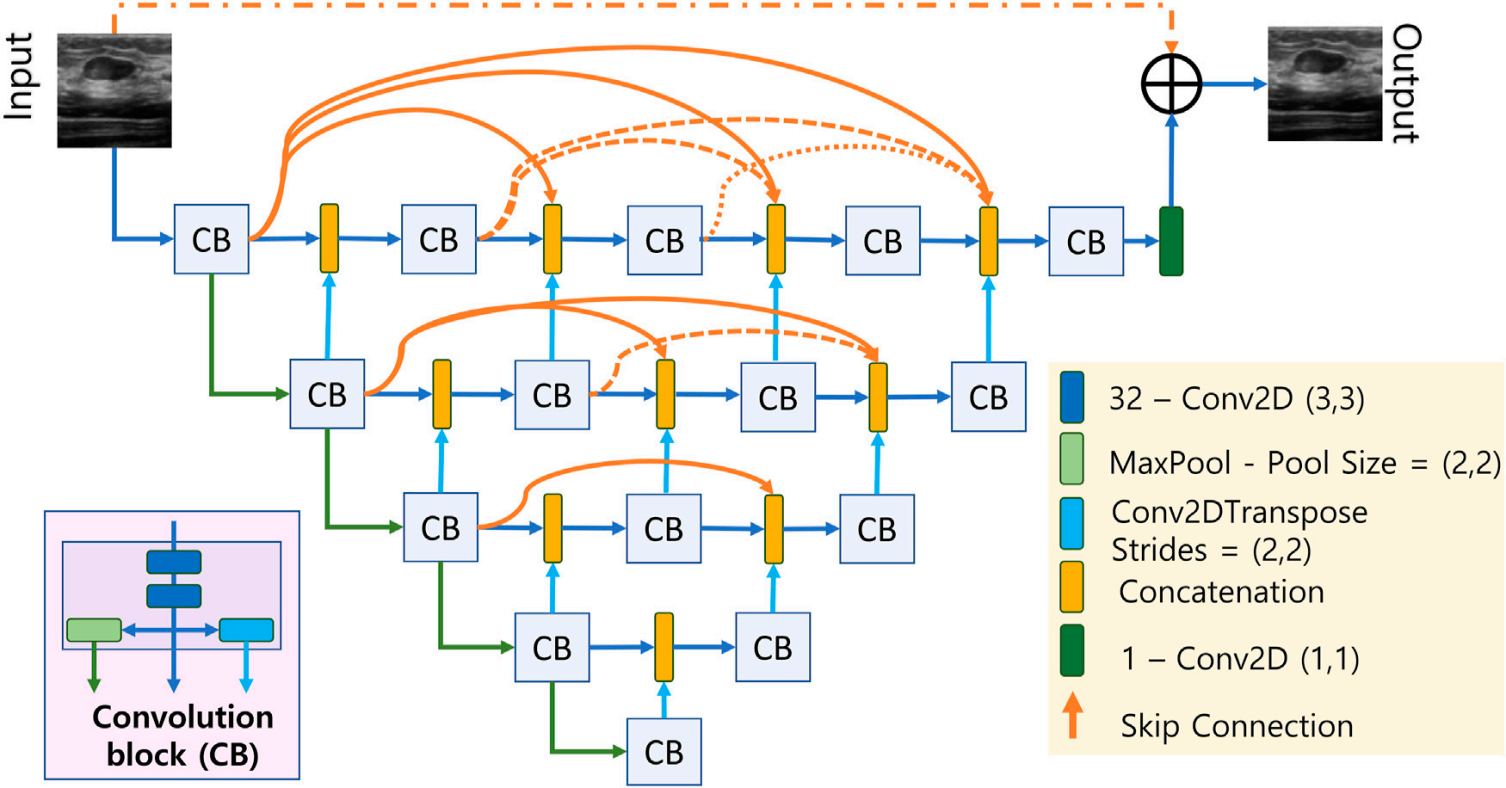
Densly connected UNET-type CNN.

The proposed deep CNN consists of a UNET-like network followed by a resolution/texture enhancement network. The architecture of the UNET-like network has a deep encoder-decoder structure where the encoder and decoder networks are densly connected by skip connections as shown in Figure 2. The short skip connections are facilitated by the concatenation blocks where the number of inputs to each concatenation block increments by a 1 as we move from input to the output which ensures the model training in an accurate and efficient manner (Huang et al., 2017). Each convolution block labelled as CB in Figure 2 contains two stacked convolution layers and results in 3 outputs; one from the stacked convolutions, one after a (2,2) maxpooling layer, and the final one from a transposed convolution layer with a stride size of 2. It can be seen in Figure 2 that the input dimensions are reduced by a factor of 2 along the encoding path and it is upsampled by the same factor by means of transpose convolution in the decoding path. After the final CB, the number of channels are reduced to 1 using a (1,1) convolution layer having only 1 filter. We also incorporate residual learning by adding the output of the final (1,1) convolution layer with the input by means of a long skip connection.

**FIGURE 3:**
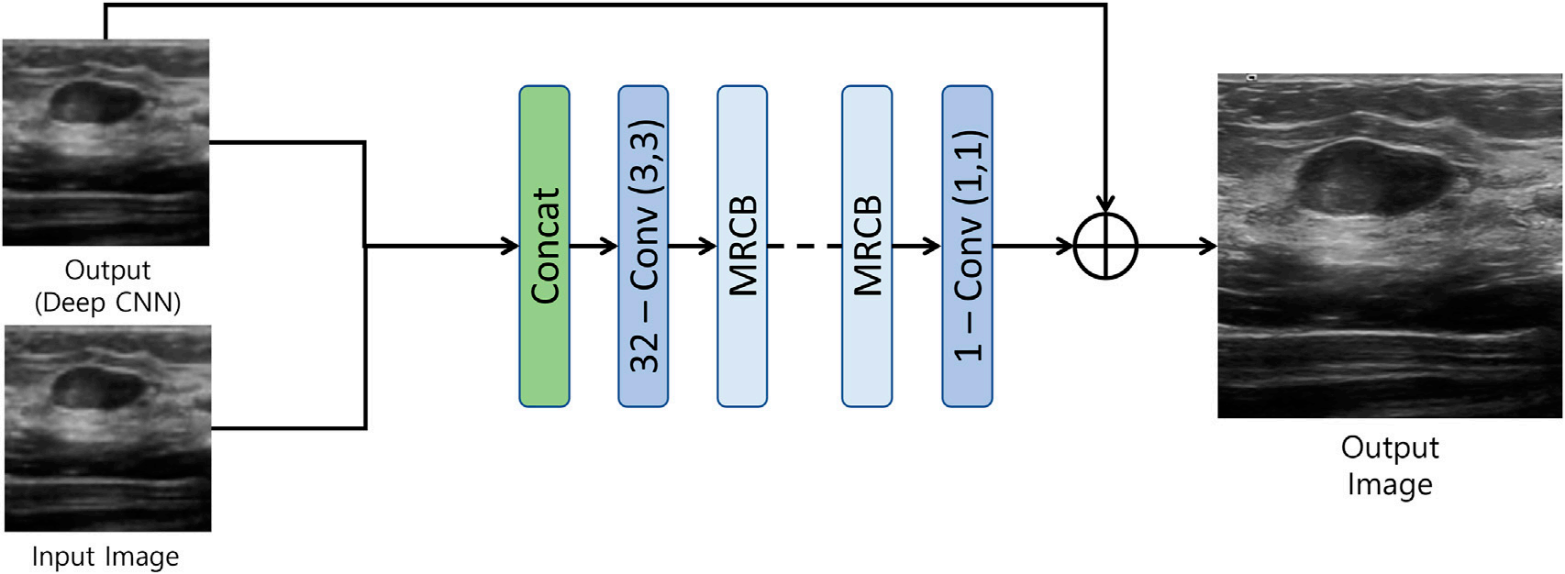
Resolution/Texture enhancement network.

**FIGURE 4:**
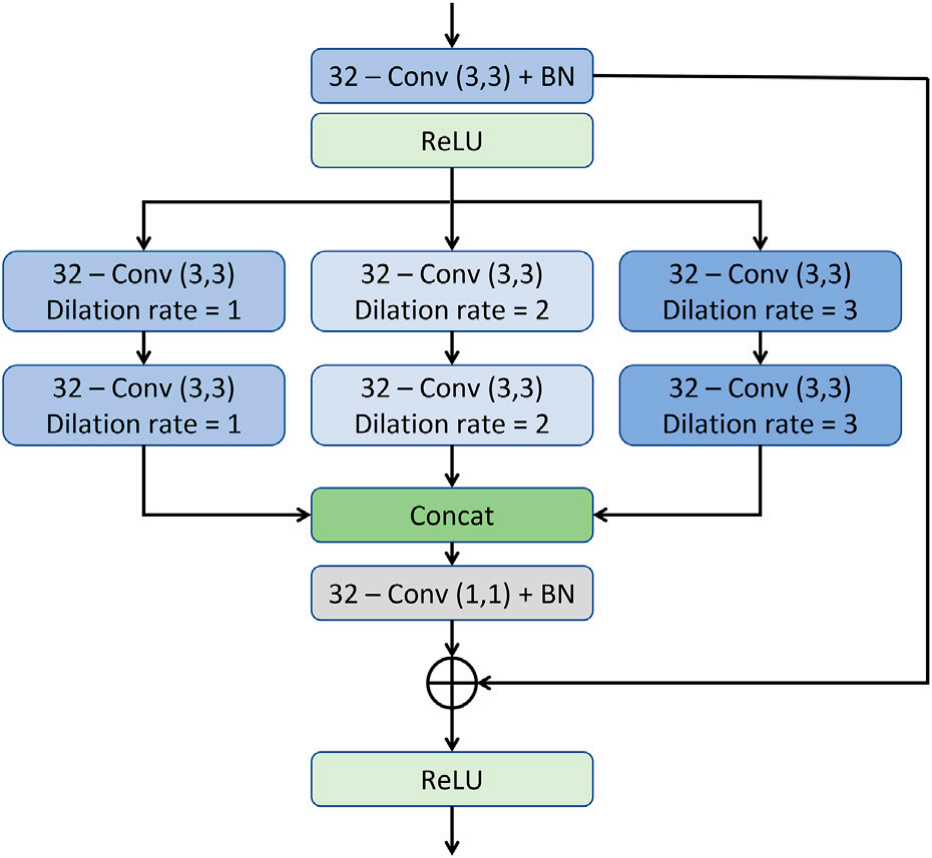
The architecture of the multi-resolution convolution block.

The output of the first model is then fed to the resolution/texture enhancement network as shown in Figure 3. This network takes two inputs; 1) the output of the densly connected UNET-type CNN, and 2) the input image. Both the inputs are concatenated and fed to a convolution layer. The output of the convolution layer is then fed to a stack of multi-resolution convolution blocks (MRCB). The architecture of the MRCB is shown in Figure 4. Since denoising models usually blur-out the high frequency texture information due to their high correlation with speckle noise, we aim to preserve the texture information using dilated convolutions in the MRCB. Varying the dilation rate varies the size of the receptive field of the convolutions and preserve texture information in the input image (Zhang et al., 2021).

**FIGURE 5:**
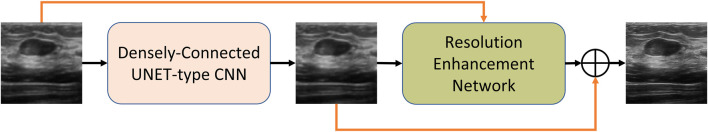
The overall block diagram of the proposed method.

As illustrated in the MRCB architecture depicted in Figure 4, the input to the MRCB is passed onto a convolution layer followed by the batch normalization and activation (ReLU) layer. The output of the activation layer is fed to stacks of 3 parallel convolutions with different dilation rates. All 3 stacks of convolution layers have different dilation rates that can ensure the extraction of the context information at 3 different scales due to the variation in the size of the receptive fields. The advantage of using different dilation configuration is also two-folds; 1) it helps improving the texture information, 2) it also helps in reducing the number of parameters of the network as compared to using the convolution layers with different filter sizes e.g. (3, 3), (5, 5), (7, 7), etc. The output of the final MRCB is forwarded to a convolution layer with a kernel size of (1,1), and the output of the final convolution layer is added with the input of the resolution enhancement network to produce the final output of the model. The overall framework of the proposed system is presented in Figure 5.

## 3 Experimental setup and results

### 3.1 Training setup

In order to assess the effect of utilizing US image formation physics knowledge in the training, we devised two distinct training schemes. In the first scheme, scheme 1: we train our network only on the real-world breast US (BUS) dataset presented in (Yap et al., 2017). The BUS dataset comprises of 163 breast ultrasound images of which, 53 images are of malignant and 110 images are of benign lesions. Since the objective of this work is to perform image enhancement (image-to-image translation), we generated label images (high resolution and low noise) using the state-of-the-art non-local low-rank (NLLR) normal filtration (Zhu et al., 2017).

For our second training scheme, scheme 2: we designed the US image formation physics information-based augmentation dataset described in section 2.1. The augmentation dataset consists of 2000 images of which 1000 images are obtained from What is a salient object (Image) and the remaining 1000 images from What is a salient object (Ground) sets in the SOD dataset (Xia et al., 2017). From here on, we will refer to the first set of 1000 images as SOD-image, and the second set as SOD-ground. The objective behind using US image formation physics into the augmentation dataset is to improve the robustness towards different speckle profiles as well as improving the generalization ability of the model. To avoid biasness in results due to particular training set choice, and to evaluate full dataset for both the training and testing purpose, we utilize a K-fold cross validation method. Since number of real ultrasound images are limited and high number of folds e.g., 10-folds limit the test-set to just 10%. Therefore herein, we performed training and testing using 5-fold cross-validation, the 5-fold validation allow comprehensive evaluation where dataset is divided into 5 equally distributed sub -sets, and for each fold, the model is first trained on four sets and remaining set is used for test. This way complete dataset is utilized for both the training and testing purposes and mean and standard deviation statistics of performance metric are reported. The proposed model was trained on *mean-squared-error* (MSE) loss optimized using Adam optimizer with a learning rate of 1*e* − 4, and trained with batch-size of 8 for 200 epochs with an early stopping patience of 50 epochs.

Besides using different speckle profiles in the augmentation dataset, we also introduced variations in the resolution of both the datasets. The resolution of the images in both the datasets was first fixed at (256 × 256). For both the training schemes, we performed resolution reduction by simply resizing the images in both the datasets to; (64 × 64) and (128 × 128), and resized both versions back to (256 × 256) resulting in 4 separate datasets having 2 different speckle profiles as well as 2 different resolution profiles.

### 3.2 Results and discussion

**FIGURE 6:**
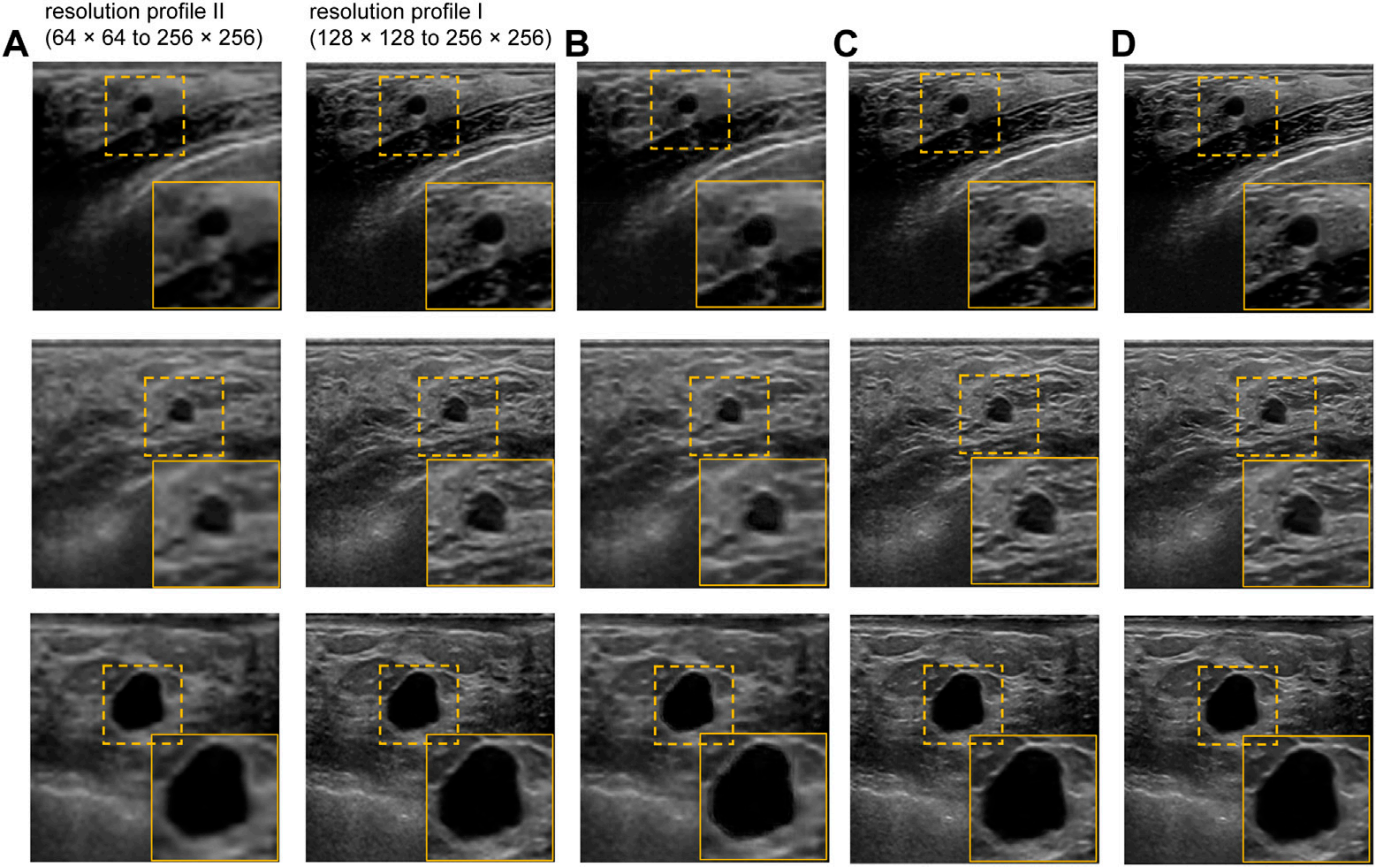
Visual results of the proposed US image enhancement model for training of the proposed network with only US image data (training Scheme 1). **(A)** Input image **(B)** training and testing on BUS dataset with resolution profile II (64 × 64 to 256 × 256) **(C)** training and testing on BUS dataset with resolution profile I (128 × 128 to 256 × 256) **(D)** Label image filtered using non-local low-rank (NLLR) normal filtration (Zhu et al., 2017).

The performance of our proposed method is evaluated qualitatively by the visual inspection of the images, and quantitatively using the standard peak signal-to-noise ratio (PSNR) and structural similarity index (SSIM) values. We also compared the performance of the proposed US image enhancement model with 3 different deep CNN models namely UNET++ (Zhou et al., 2018), UNET (Ronneberger et al., 2015), and NMB-TCB (Zhang et al., 2021). In order to evaluate the improvement in resolution, we also calculated contrast (Tang et al., 2009) using generalized contrast-to-noise ratio (GCNR) (Rodriguez-Molares et al., 2018) for each training scheme and respective datasets. The visual results of training scheme 1, where we train and test our proposed method with only real-world US images with different noise and resolution profiles are presented in Figure 6. It is evident from the Figure 6C that the best visual performance for training scheme 1 is obtained on the BUS dataset with resolution profile I.

**FIGURE 7:**
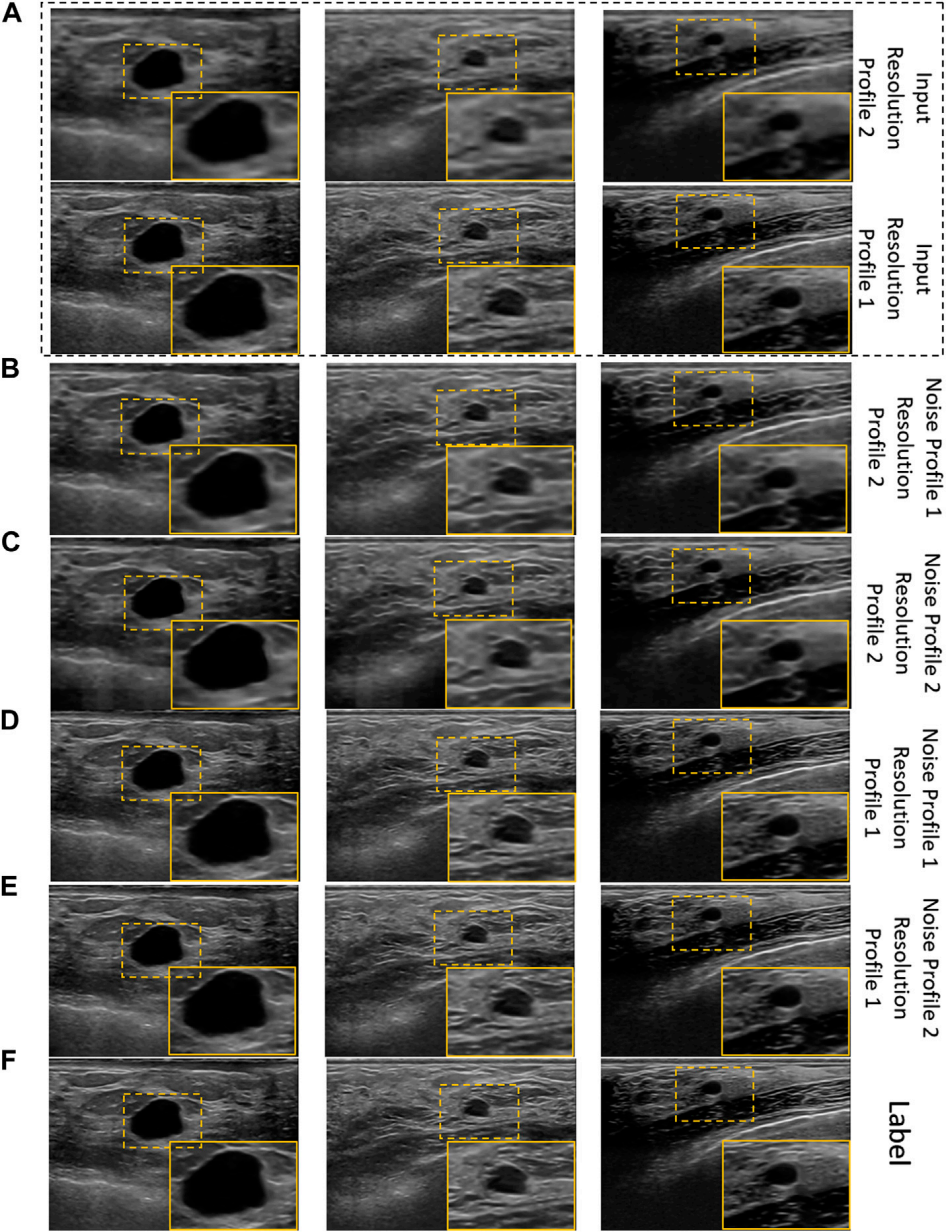
Visual results of the proposed US image enhancement model for training and testing of the proposed network with US image data as well as US image formation physics informed augmentation dataset (training scheme 2). **(A)** Input image **(B)** training and testing on noise profile I (Rayleigh noise variance = 0.2, Gaussian filter standard deviation = 0.8) with resolution profile II (64 × 64 to 256 × 256) **(C)** training and testing on noise profile II (Rayleigh noise variance = 0.1, Gaussian filter standard deviation = 0.7) with resolution profile II (64 × 64 to 256 × 256) **(D)** training and testing on noise profile I (Rayleigh noise variance = 0.2, Gaussian filter standard deviation = 0.8) with resolution profile I (128 × 128 to 256 × 256) **(E)** training and testing on noise profile II (Rayleigh noise variance = 0.1, Gaussian filter standard deviation = 0.7) with resolution profile I (128 × 128 to 256 × 256) **(F)** Label image filtered using non-local low-rank (NLLR) normal filtration (Zhu et al., 2017).

The qualitative results of cross-validation on training scheme 2 are presented in Figure 7. It can be observed that the proposed model performs relatively better with the resolution profile I where the resolution of the input images are reduced by a factor of 4. Observing the outputs of other noise and resolution profiles closely also reveals that the noise and texture has improved but is not as promising as the visual results in Figure 6C and Figure 7E for training scheme 1, 2, respectively.

### 3.2.1 Analysing the effect of proposed US image formation physics-informed dataset augmentation

To investigate the performance of the proposed method as well as the effectiveness of the proposed US image formation physics-informed dataset augmentation, we designed an ablation study for our proposed method. First, we evaluate the performance of our proposed method with US image formation physics knowledge introduction in the augmentation dataset. Table 2 presents the PSNR, SSIM, and GCNR results for the ablation study on the effect of the proposed US image formation physics informed augmentation in the training of the proposed model. We present the results for a specific noise and resolution profile (noise profile II, resolution profile I) in order to best describe the effect of using US image formation knowledge in the training of an image enhancement model. It can be easily observed that the PSNR, SSIM, and GCNR have improved with the proposed training setup where we suggested to incorporate US image formation physics knowledge in to the model training.

**TABLE 2:** PSNR, SSIM, and GCNR result for the ablation study on the effect of US image formation physics informed data augmentation. (Input PSNR = 32.1392 ± 2.7026, Input SSIM = 0.9279 ± 0.0252, Input GCNR = 0.9974 ± 0.0017).

Training Method	Output PSNR	Output SSIM	Output GCNR
Without Augmentation	32.6381 ± 2.1588	0.9198 ± 0.0279	0.9958 ± 0.0030
With Augmentation	33.0270 ± 3.0317	0.9408 ± 0.0247	0.9990 ± 0.0008

### 3.2.2 Analysing model performance on different resolution profiles

We designed the second ablation study to investigate the effect of different resolution profiles on the model performance. In this ablation study, we only present the results of training with US image formation physics-based augmentation only to have a better understanding of the effect of different resolution profiles on the model performance. This ablation study is summarized in Table 3. It is evident from the ablation study presented in Table 3 that there is 2.76%, 1.39%, and 0.16% improvement in PSNR, SSIM, and GCNR respectively for the resolution (128 × 128). For the resolution of (64 × 64), there is 3.48%, 5.97%, and 0.302% improvement in PSNR, SSIM, and GCNR, respectively. This shows the robustness of our model against different resolution profiles.

**TABLE 3:** PSNR, SSIM, and GCNR result for the ablation study on the effect of different resolution profiles on the model performance. (128 **×** 128 Input PSNR = 32.1392 ± 2.7026, Input SSIM = 0.9279 ± 0.0252, Input GCNR = 0.9974 ± 0.0017) (64 **×** 64 Input PSNR = 26.0071 ± 2.3083, Input SSIM = 0.7098 ± 0.0761, Input GCNR = 0.9936 ± 0.0039).

Resolution Profile	Output PSNR	Output SSIM	Output GCNR
128 × 128	33.0270 ± 3.0317	0.9408 ± 0.0247	0.9990 ± 0.0008
64 × 64	26.9112 ± 2.3025	0.7522 ± 0.0635	0.9966 ± 0.0026

### 3.2.3 Analysing the robustness of proposed method against different noise levels

The third ablation study was designed to assess the performance of our proposed method with variation in the noise profiles. Table 4 presents the findings of this ablation study. It can be seen from the ablation study that there is 3.48%, 5.97%, and 0.302% improvement in PSNR, SSIM, and GCNR, respectively for the noise profile I. For the noise profile II, there is 2.87%, 5.64%, and 0.44% improvement in PSNR, SSIM, and GCNR respectively. It can be concluded from the ablation study that the best performance combination is to train the model with US image formation physics knowledge-based augmentation with noise profile I and a resolution of 64 × 64.

**TABLE 4:** PSNR, SSIM, and GCNR result for the ablation study on the effect of different resolution profiles on the model performance. (Noise Profile I (Rayleigh noise variance = 0.2, Gaussian filter standard deviation = 0.8). Noise Profile II (Rayleigh noise variance = 0.1, Gaussian filter standard deviation = 0.7). Input PSNR = 26.0071 ± 2.3083, Input SSIM = 0.7098 ± 0.0761, Input GCNR = 0.9936 ± 0.0039).

Noise Profile	Output PSNR	Output SSIM	Output GCNR
I	26.9112 ± 2.3025	0.7522 ± 0.0635	0.9966 ± 0.0026
II	26.7547 ± 2.3758	0.7498 ± 0.0611	0.9980 ± 0.0017

### 3.2.4 Analysing the robustness of proposed method against different contrast levels

**FIGURE 8:**
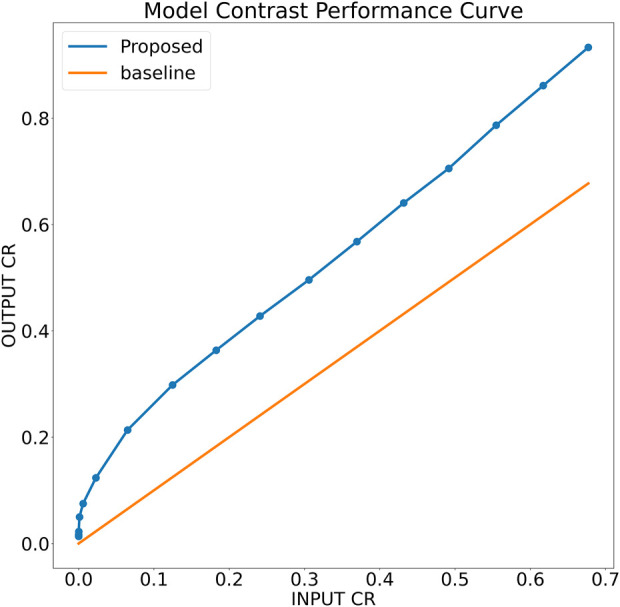
Contrast recovery curve for the proposed model trained on noise profile I (Rayleigh noise variance = 0.2, Gaussian filter standard deviation = 0.8)

To further analyze the robustness of the proposed model, a simulation phantom is designed that consist of an arbitrary shape of hyper-echoic region and hypo-echoic background. Using the simulated phantom, input images of different degradation levels were generated. In particular, in a 256 × 256 clean image first a Rayleigh noise is added followed by compression (256 × 256 → 64 × 64) and re-expansion (64 × 64 → 256 × 256) steps and finally the low-resolution noisy version is further blurred with the help of Gaussian filtration. The noise variance is varied from 0.25 to 1.95 units with the step-size of 0.05 units, for each noise level, the Gaussian blurring standard deviation is also varied accordingly staring from 1.25 to 2.95 units with the step-size of 0.05 units, respectively. The noisy images are filtered using the proposed model trained on noise profile I (Rayleigh noise variance = 0.2, Gaussian filter standard deviation = 0.8) and contrast-recovery (CR) scores are compared for input and output images. A comparison of baseline and proposed contrast recovery curves is shown in Figure 8. The Python colab-notebook implementation of the proposed algorithm along with the simulation setup used for this experiment is available at author’s GitHub page (https://github.com/Shujaat123/MUSI_Enhancement_TCMR_CNN).

### 3.2.5 Analysing model sensitivity over hyper-parameters

In order to evaluate the performance variation with different hyperparameters in the proposed method, an ablation study to assess the effect of various convolution kernel sizes, number of kernels, inference time, and the effect of different pooling techniques is also presented. In this ablation study, we first present the effect of Max Pooling and Average Pooling on the performance of the proposed technique. The results of this ablation study are presented in Table 5. The results in the table present the mean output PSNR results of the three datasets used in this study. It can be observed from the mean output PSNR results from the model with Maxpooling layer instead of average pooling layer can result in better output PSNR with various model hyperparameter combinations.

**TABLE 5:** Mean PSNR results for the ablation study on the effect of different pooling techniques on the model performance. (Mean input PSNR = 13.3726 ± 0.9174).

Pooling Technique	Kernel Size	No. of Kernels	Mean Output PSNR
Average Pooling	3 × 3	16	28.2198 ± 2.7456
		32	28.6411 ± 2.9074
		64	28.9790 ± 3.0236
	5 × 5	16	28.7586 ± 2.8746
		32	29.2008 ± 2.7406
		64	29.1355 ± 2.8385
Max Pooling	3 × 3	16	29.1414 ± 2.7476
		32	29.1743 ± 2.7404
		64	29.6820 ± 2.7956
	5 × 5	16	29.0112 ± 2.7417
		32	29.4757 ± 2.7501
		64	29.7968 ± 2.8557

The ablation study results presented in Table 5 also reveal that using maxpooling with 64 kernels of size 5 × 5 result in the best mean output PSNR. However, using this configuration of hyperparameters can be computationally expensive. Table 6 present the number of parameters and mean inference time for the various hyperparameter configurations. All experiments were conducted on a Google colab-pro notebook using Intel(R) Xeon(R) CPU @ 2.20 GHz processor and NVIDIA Tesla K80 GPU. For full ablation study study it took less than 3 days to complete. The total training time varies with the early stopping condition, batch-size and input image resolution and noise levels, etc. Furthermore, the training and testing time also varies with the size of model, however, for Max and Average pooling, the computation time was statistically identical, whereas for number of channels it increases linearly, and for kernel-size, it increases exponentially. A detailed ablation study showing inference time for each configuration is shown in Table 5. Although the ablation study presented in Table 5 suggests the use of the model hyperparameter configuration presented in the last row (Pooling:Max, Kernel size:5 × 5, Number of kernels: 64), but this configuration results in 11.05× more parameters compared to the configuration (Pooling:Max, Kernel size:3 × 3, Number of kernels: 32), as presented in Table 6. Also, the mean inference time of the former configuration is 2.428× higher than that of the latter with only 2.089% degradation in mean output PSNR.

**TABLE 6:** Mean inference time and model parameters for various hyperparameter configurations.

Kernel Size	No. of Kernels	Inference Time (Seconds)		Model Parameters
		CPU	GPU	
3 × 3	16	0.390	0.016	107,650
	32	0.978	0.021	427,266
	64	3	0.045	1,702,402
5 × 5	16	1	0.033	297,090
	32	2	0.051	1,182,978
	64	9	0.121	4,721,154

### 3.2.6 Comparison with contemporary deep learning-based solutions

The PSNR and SSIM results for both the training schemes the noise profiles, and the resolution profiles were compared with UNET++ (Zhou et al., 2018), UNET (Ronneberger et al., 2015), and NMB-TCB (Zhang et al., 2021). For fair comparison, we perfomed 5-fold cross validation for these models with the same learning rate, number of epochs, and early stopping patience for training as used with proposed model and report mean PSNR and SSIM results. Table 7 presents a comparison of the mean PSNR and SSIM values for the datasets, and also enlists the GCNR results for the SOD-Ground dataset. Although, the PSNR values for BUS dataset do not show a significant improvement, it can be seen that using the proposed method and the US image formation physics knowledge in the training can bring noticeable improvements in the SSIM values for BUS dataset. Moreover, it is also evident from the comparative results that the proposed method outperforms all the other methods in-terms of both PSNR and SSIM. It can also be seen that the PSNR and SSIM values of the proposed method on the SOD datasets also improved greatly using the proposed method which advocates the robustness of our proposed method.

**TABLE 7:** Comaprison of PSNR, SSIM, and GCNR results. The mean input PSNR for BUS dataset is 26.0071 ± 2.3083 dB, SOD-image dataset is 12.1587 ± 0.7839 dB, and SOD-Ground dataset is 12.5272 ± 0.8243 dB. The mean input SSIM for BUS dataset is 0.7098 ± 0.0761 dB, SOD-image dataset is 0.5570 ± 0.1205 dB, and SOD-Ground dataset is 0.1556 ± 0.1451 dB. The mean input GCNR for SOD-Ground dataset is 0.9936 ± 0.0039.

Method	Dataset	Output PSNR	Output SSIM	Output GCNR
NMB-TCB (Zhang et al., 2021)	BUS	22.9819 ± 2.5252	0.6171 ± 0.0661	—
	SOD-Image	11.2623 ± 2.2623	0.3052 ± 0.1153	—
	SOD-Ground	11.3659 ± 1.0254	0.1448 ± 0.1424	0.9835 ± 0.0101
UNET (Ronneberger et al., 2015)	BUS	26.8307 ± 2.2531	0.7434 ± 0.0645	—
	SOD-Image	12.0692 ± 1.0108	0.3507 ± 0.0908	—
	SOD-Ground	11.8374 ± 0.7337	0.1265 ± 0.1192	0.9880 ± 0.0135
UNET++ (Zhou et al., 2018)	BUS	26.1576 ± 2.1916	0.7111 ± 0.0629	—
	SOD-Image	11.5688 ± 0.9111	0.3056 ± 0.1073	—
	SOD-Ground	12.1533 ± 0.7603	0.1426 ± 0.1370	0.9849 ± 0.0141
Proposed	BUS	26.9112 ± 2.3025	0.7522 ± 0.0635	—
	SOD-Image	25.5275 ± 2.9712	0.6946 ± 0.1267	—
	SOD-Ground	32.4719 ± 2.6179	0.8785 ± 0.0766	0.9966 ± 0.0026

We also calculated GCNR for the SOD-ground dataset to evaluate the improvement in contrast in the output images of the proposed method compared to the other methods. GCNR is an image quality index based on the overlap area of the probability density function inside and outside the target area (Rodriguez-Molares et al., 2018). The GCNR results are presented in Table 7. It is evident from the table that the proposed method outperforms all other deep CNN models in-terms of GCNR. To summarize, it can be observed from the ablation studies and comparative results that the proposed deep CNN-based US image enhancement method can not only perform speckle noise reduction, but can also preserve the texture information by leveraging the fusion of receptive fields of different sizes in the resolution enhancement network. It can also be concluded from the results that augmenting US image formation physics informed data with the real-world US datasets can improve the performance as well as generalization ability of deep learning-based US image enhancement methods.

### 3.2.7 Comparison with conventional speckle-denoising methods

To provide a comparison with conventional ultrasound speckle denoising techniques, different algorithms are selected to represent diverse filtration techniques, herein we implemented three popular methods namely Probabilistic Patch-Based filter (PPB) (Deledalle et al., 2009), speckle-reducing anisotropic diffusion (SRAD) algorithm (Yu and Acton, 2002; Aja-Fernández and Alberola-López, 2006) and image filtered using non-local low-rank (NLLR) normal filtration (Zhu et al., 2017) and compare them with the proposed model trained on noise profile II (Rayleigh noise variance = 0.1, Gaussian filter standard deviation = 0.7). The codes for PPB and NLLR are downloaded from their official sites while for SRAD the official MATLAB built-in functions of *specklefilt* is used. For SRAD, default settings were used whereas for PPB, the noise levels are set to 2, and filter size of 3 × 3 was used, while for NLLR, the same configuration i.e., beta = 10, and H = 5 is used for generating ground truths is used here as well.

**FIGURE 9:**
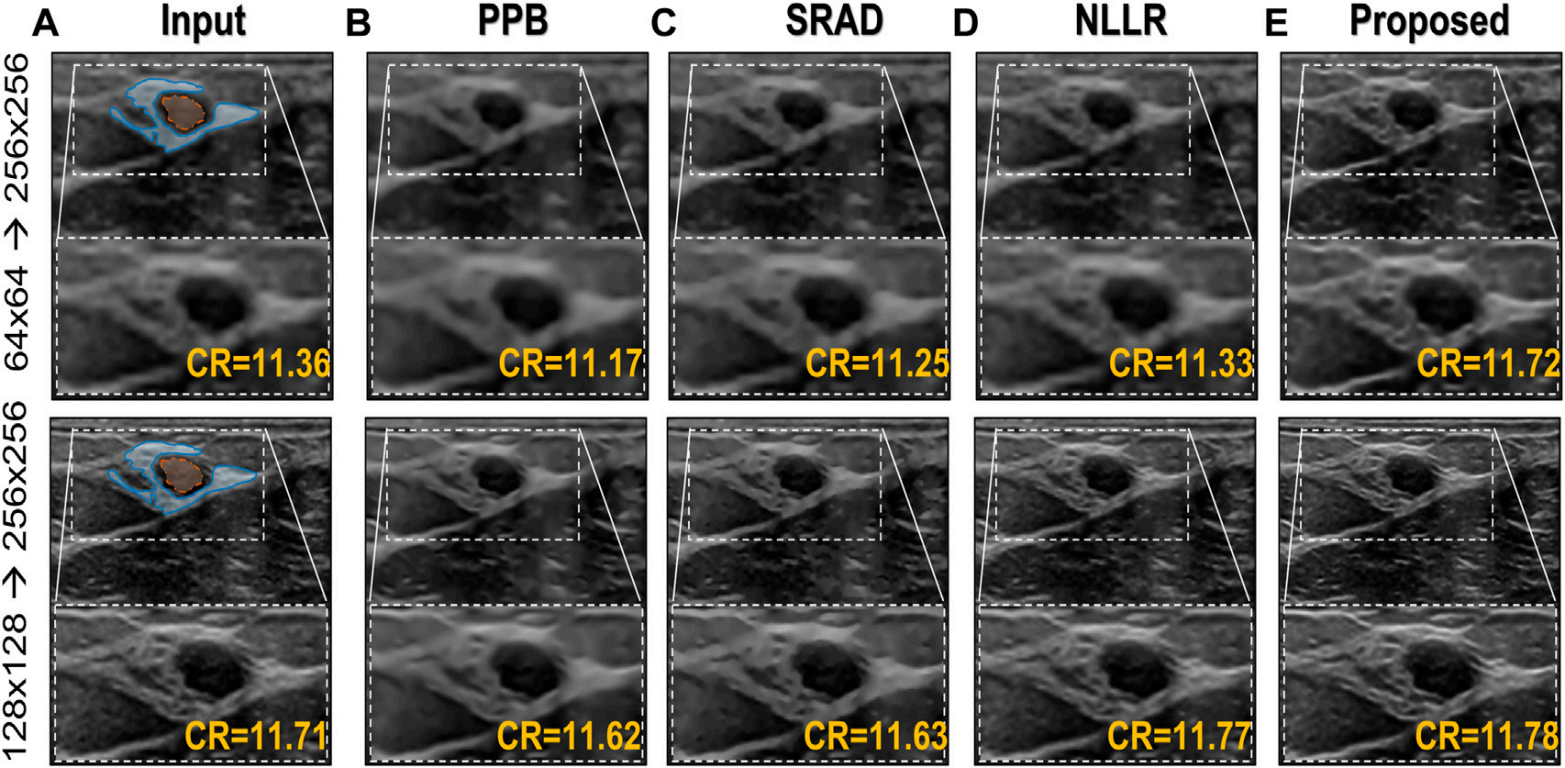
Comparison of the proposed image enhancement method with conventional speckle denoising methods. **(A)** Input image **(B)** Probabilistic Patch-Based filter (PPB) (Deledalle et al., 2009) filtered image using 2 levels of 3 × 3 filters **(C)** results of speckle-reducing anisotropic diffusion (SRAD) algorithm (Yu and Acton, 2002; Aja-Fernández and Alberola-López, 2006) **(D)** image filtered using NLLR (Zhu et al., 2017), and **(E)** Proposed model trained on noise profile II (Rayleigh noise variance = 0.1, Gaussian filter standard deviation = 0.7)

As mentioned earlier, a major limitation of conventional image denoising methods is that they are designed to remove specific noise only, and do not improve the overall image quality e.g., resolution, contrast, etc. From the results in Figure 9 it can be seen that the proposed method does not only reduce speckle but also improves the overall quality and enhances the anatomical structural detail and overall contrast. The contrast-recovery (CR) scores for each method are shown with their corresponding results. For contrast-recovery calculation mean pixel intensities of two *regions-of-interest* (ROIs) are compared, the ROIs are shown in blue/orange masked region of input images.

**FIGURE 10:**
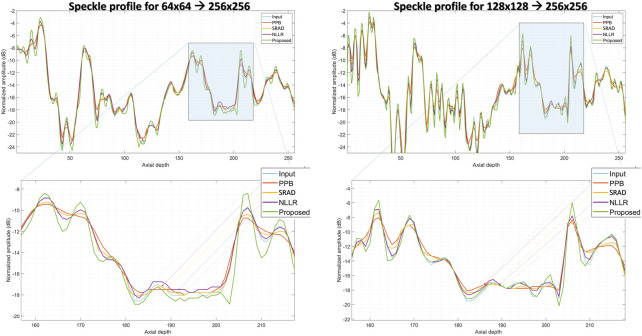
Comparison of speckle profile for two resolution settings. (top):speckle profile from the lateral mid-point of *in-vivo* sample shown in Figure 9, (bottom):zoomed-in view of the highlighted region shown in (top) figures.

In comparison, the Probabilistic Patch-Based filter (PPB) dramatically reduces the speckle, however, it also smooths out the structural details. On the other hand the speckle-reducing anisotropic diffusion (SRAD) algorithm preserves edges but also introduces blurring specially in low-resolution case. Although, the NLLR shows relatively better performance in terms of preserving structural detail and reduce noise, however, none of the aforementioned classical methods improve spatial information (which is expected) because they are not designed to perform that task. The ability to improve the overall quality both in terms of nose reduction and resolution enhancement makes our image enhancement scheme unique, which suppresses the noise to improve contrast but doesn’t compromise on resolution. Moreover, to compare resolution gain and edge preservation, the axial speckle profile is plotted from the lateral mid-point of images in Figure 10. The speckle profile results for both resolution settings shows a noticeable resolution gain.

## 4 Conclusion

In this paper, we proposed a deep CNN-based ultrasound image enhancement method. Since ultrasound images usually have low-resolution and high noise content such as speckle noise. The proposed deep CNN architecture is divided into two parts; first, a deep encoder-decoder or a UNET-like model with densly connected skip pathways aim to remove the speckle noise. This noise suppression network is followed by a resolution enhancement network which utilizes several dialted convolutions as well as residual learning to enhance the image quality by capturing the texture/content information. We propose to use the fusion of different dilation configurations in order to preserve high frequency/texture information by varying the size of the receptive fields. Besides the deep CNN network, we also propose to leverage ultrasound image formation physics to generate an augmentation dataset to aid the training as well as improve the generalization of the deep CNN model. The experimental results also showcase the superiority of the proposed method for image enhancement both in-terms of visual quality as well as the PSNR, SSIM, and GCNR results.

## Data Availability

Publicly available datasets were analyzed in this study. The BUS dataset data can be found here: http://www2.docm.mmu.ac.uk/STAFF/m.yap/dataset.php. The salient object detection (SOD) dataset can be found here: http://cvteam.net/projects/CVPR17-ELE/ELE.html.
