# Supplementary figures and images for: Medical ultrasound image speckle reduction and resolution enhancement using texture compensated multi-resolution convolution neural network
Source: Front Physiol. 2022 Nov 14;13:961571. doi: 10.3389/fphys.2022.961571 (PMC9702358; doi:10.3389/fphys.2022.961571)

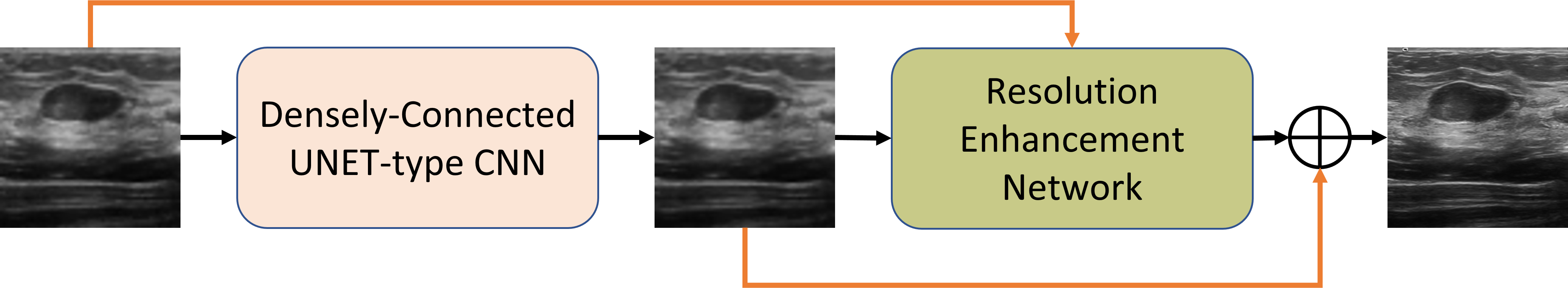

Supplement: Supplementary file 1 [file Image5.PNG]

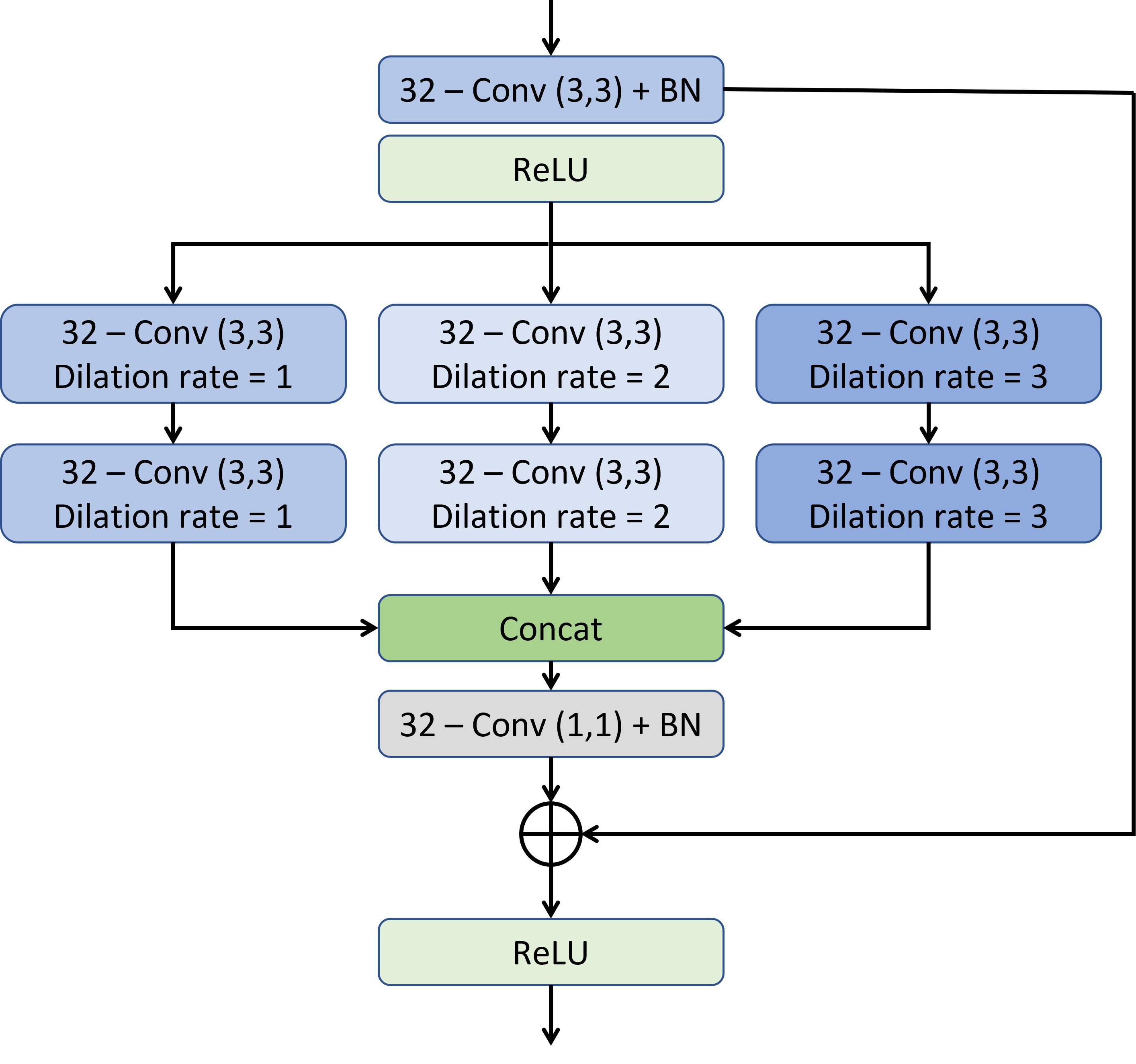

Supplement: Supplementary file 2 [file Image4.PNG]

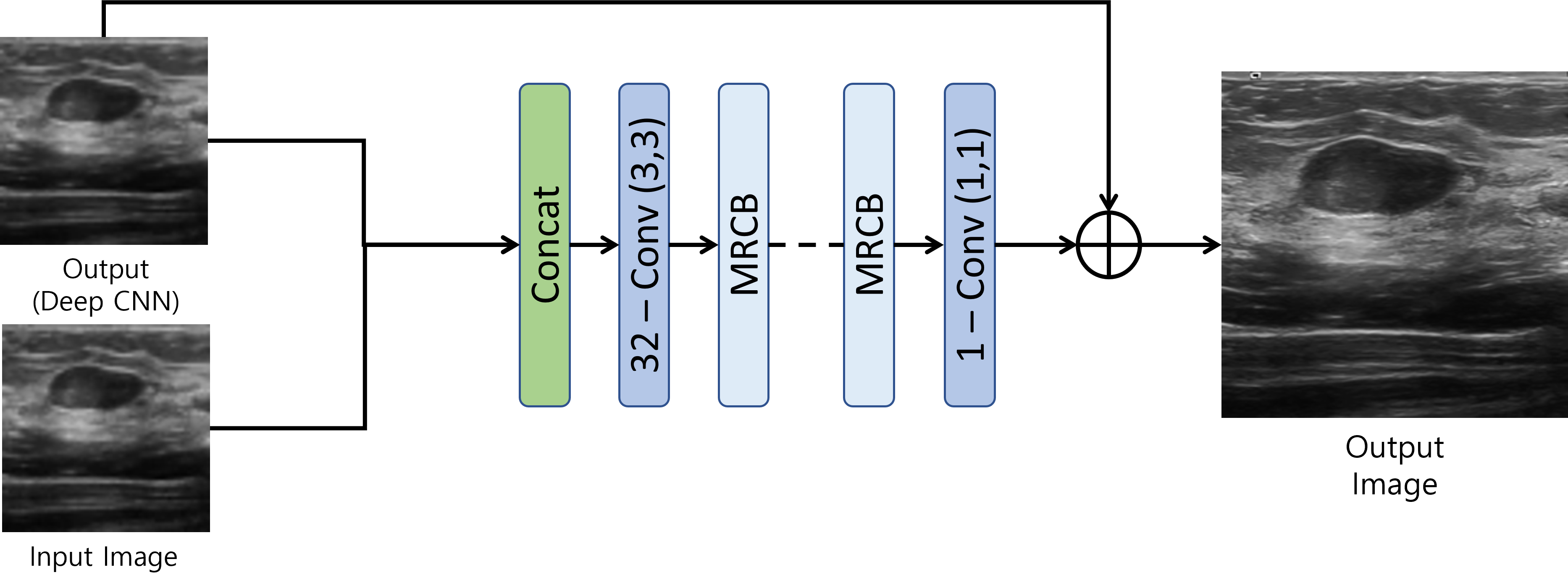

Supplement: Supplementary file 3 [file Image7.PNG]

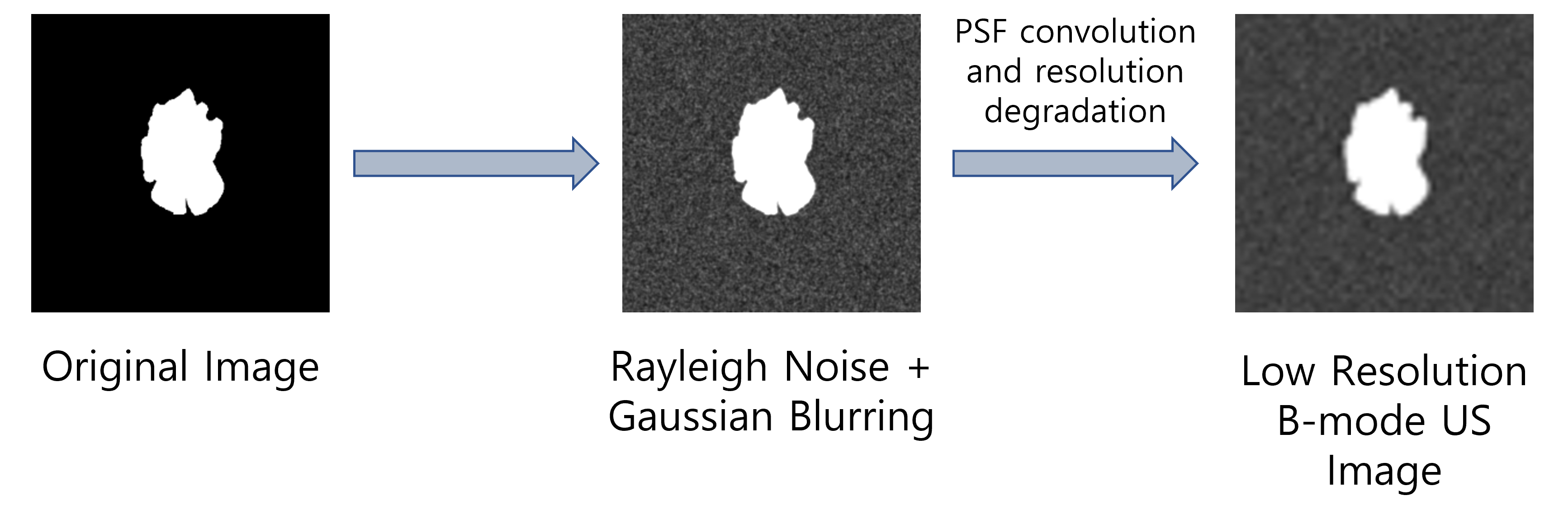

Supplement: Supplementary file 4 [file Image2.PNG]

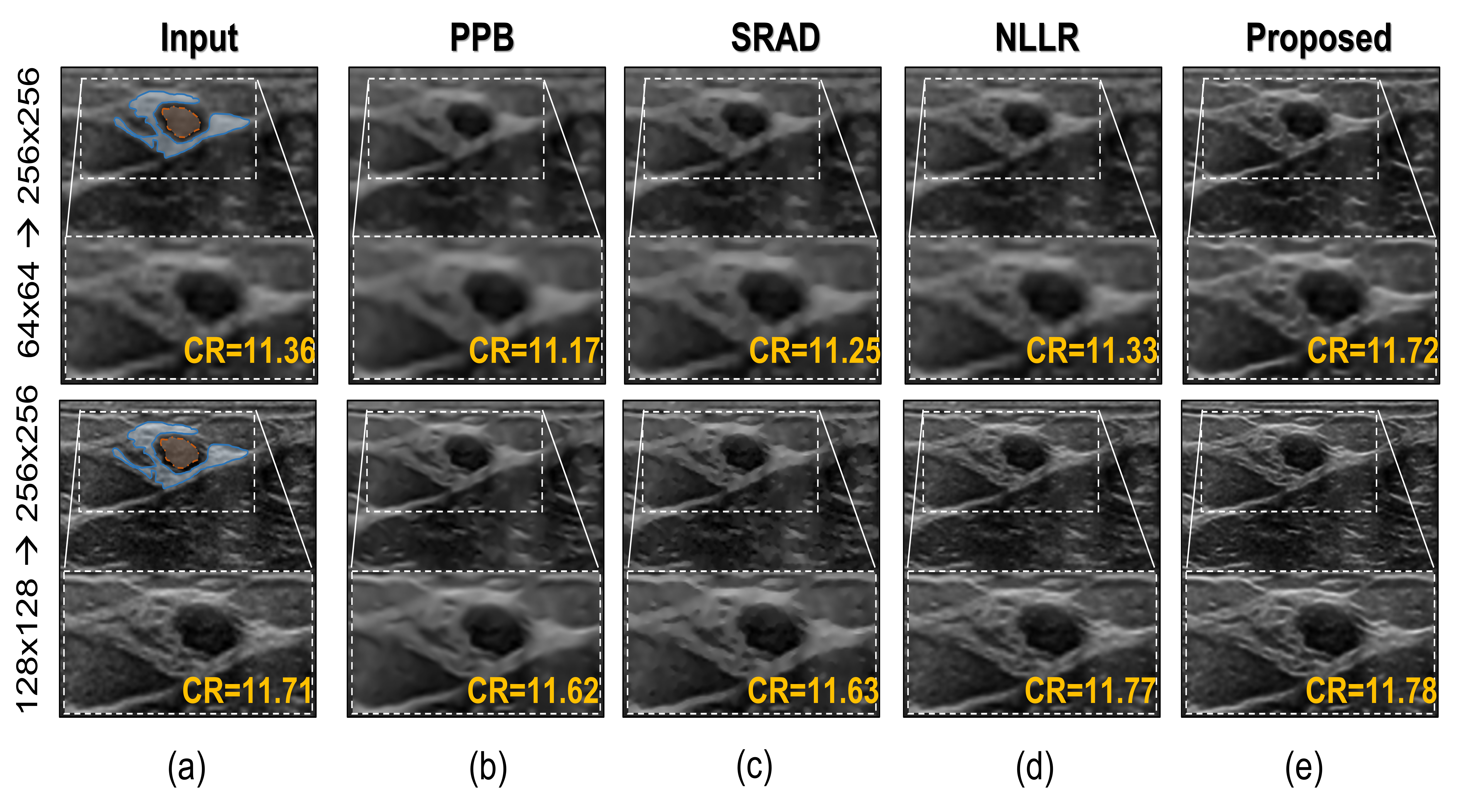

Supplement: Supplementary file 5 [file Image1.PNG]

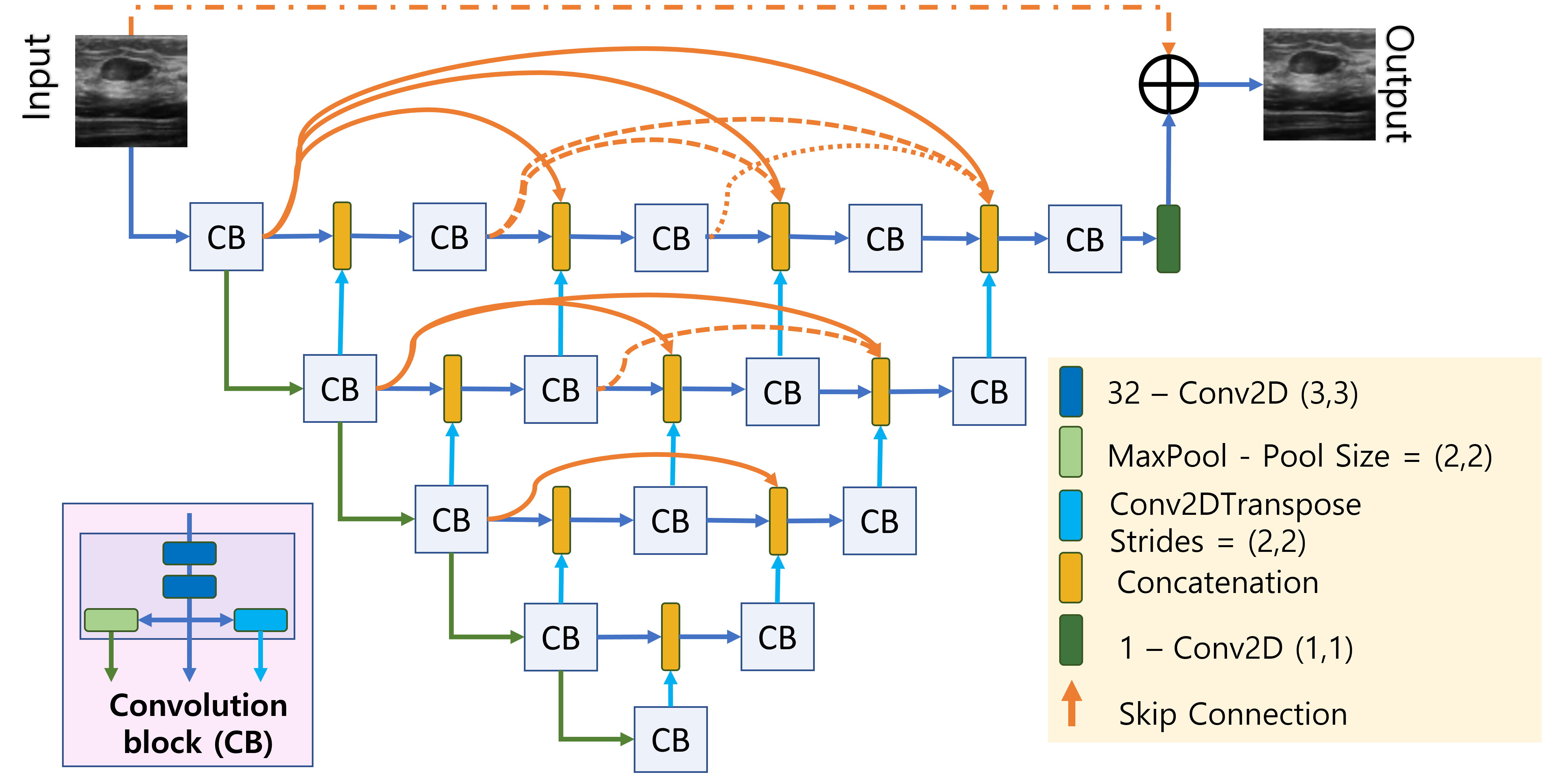

Supplement: Supplementary file 6 [file Image8.PNG]

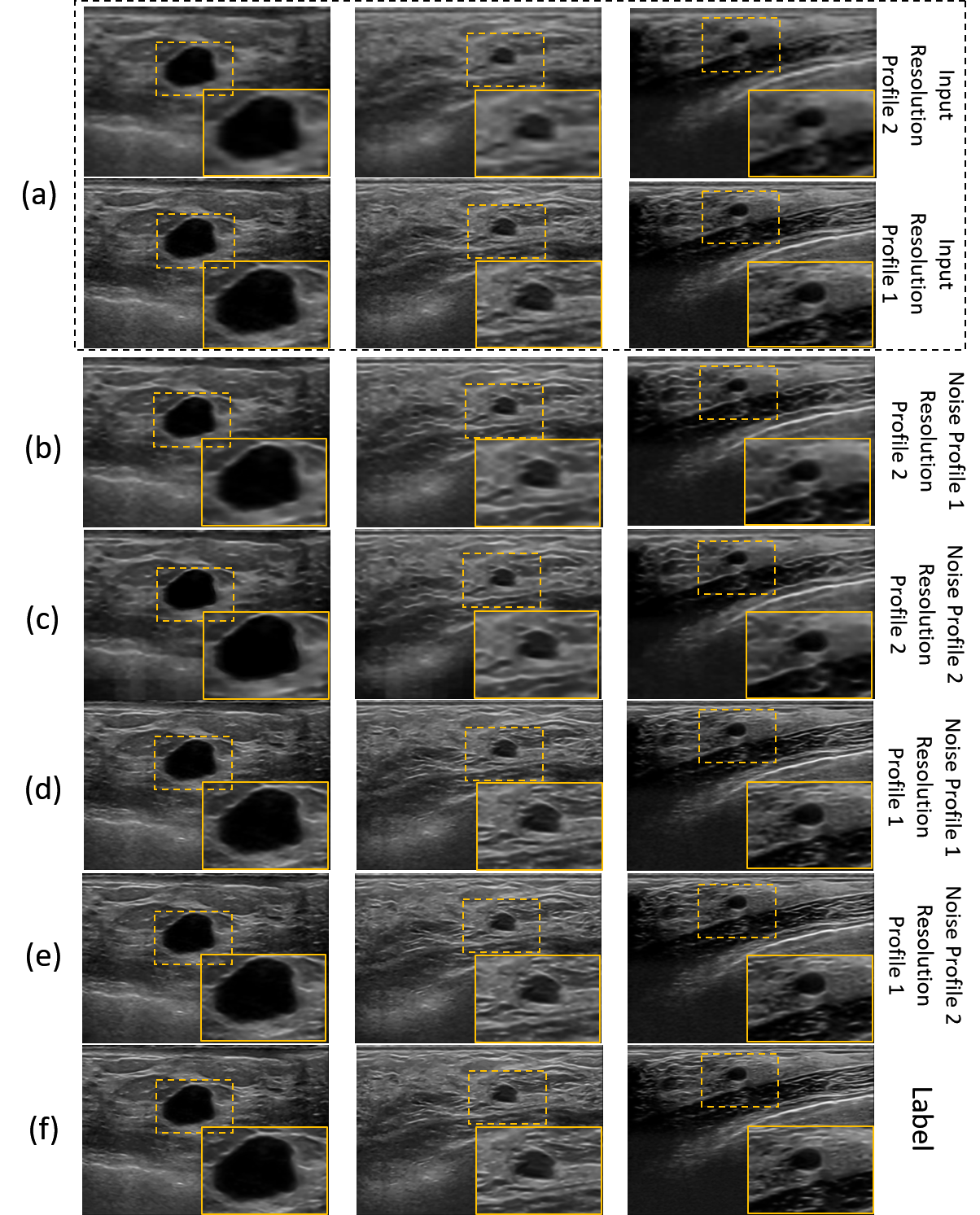

Supplement: Supplementary file 7 [file Image9.PNG]

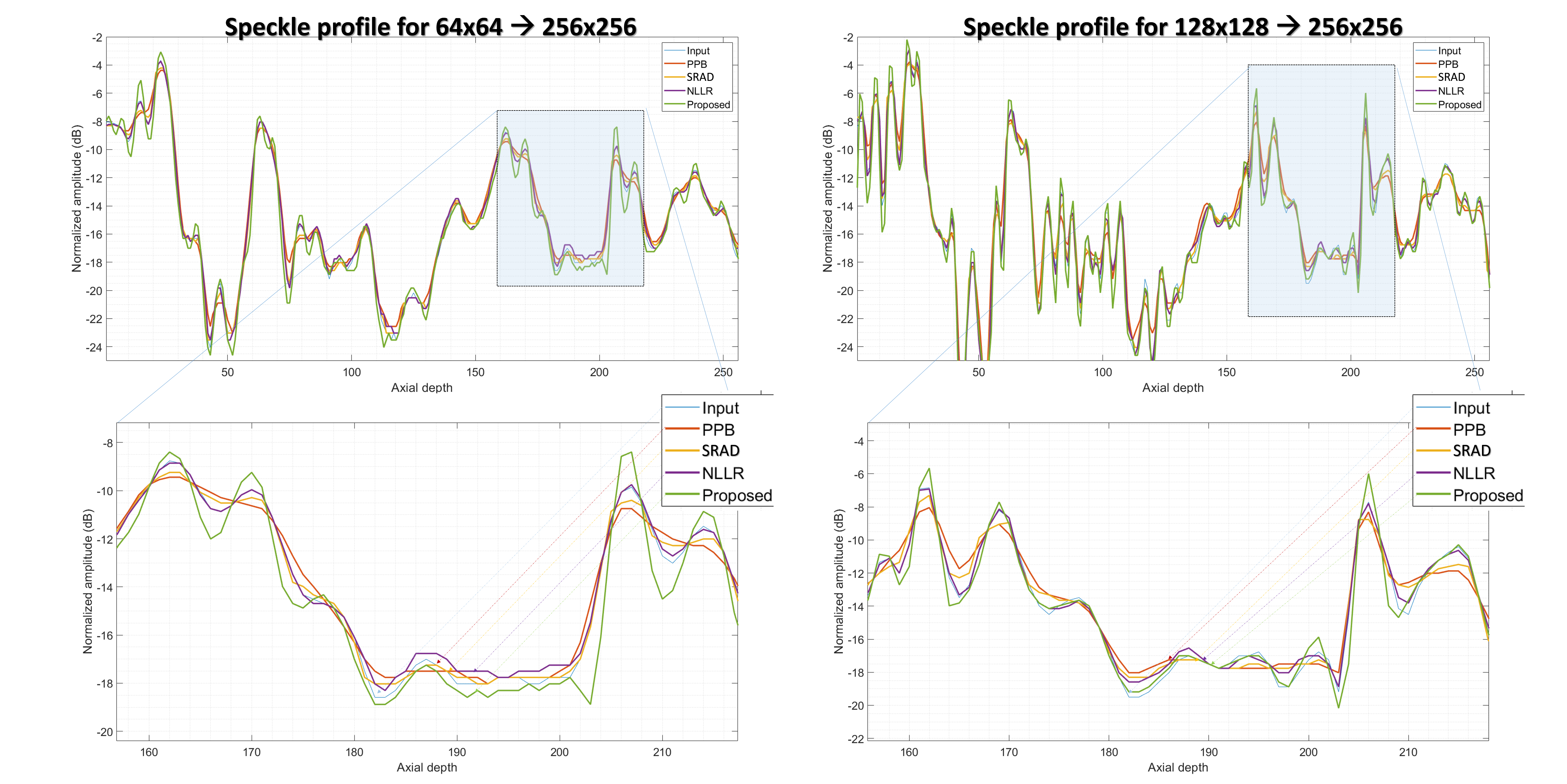

Supplement: Supplementary file 8 [file Image6.PNG]

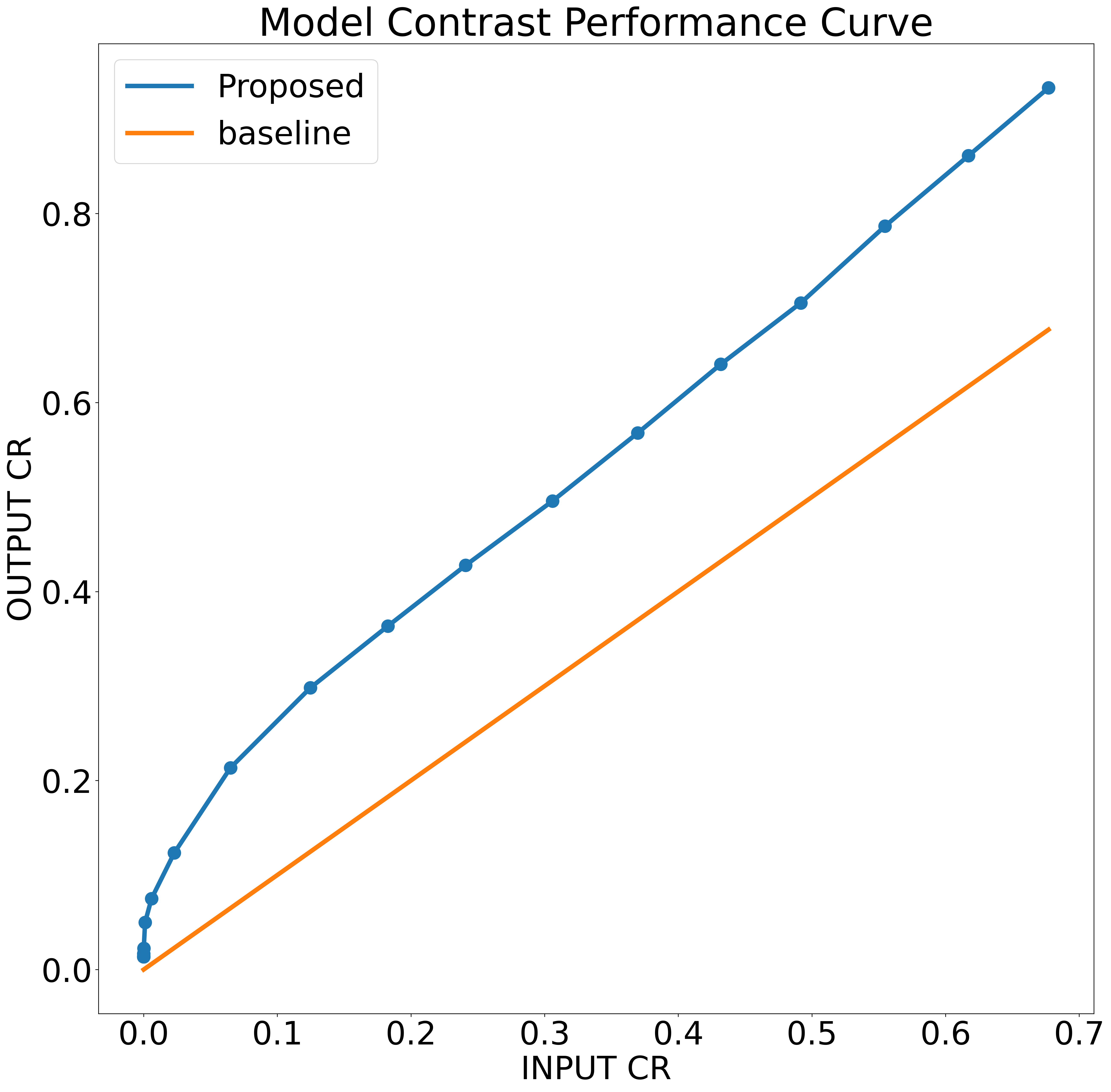

Supplement: Supplementary file 9 [file Image3.PNG]

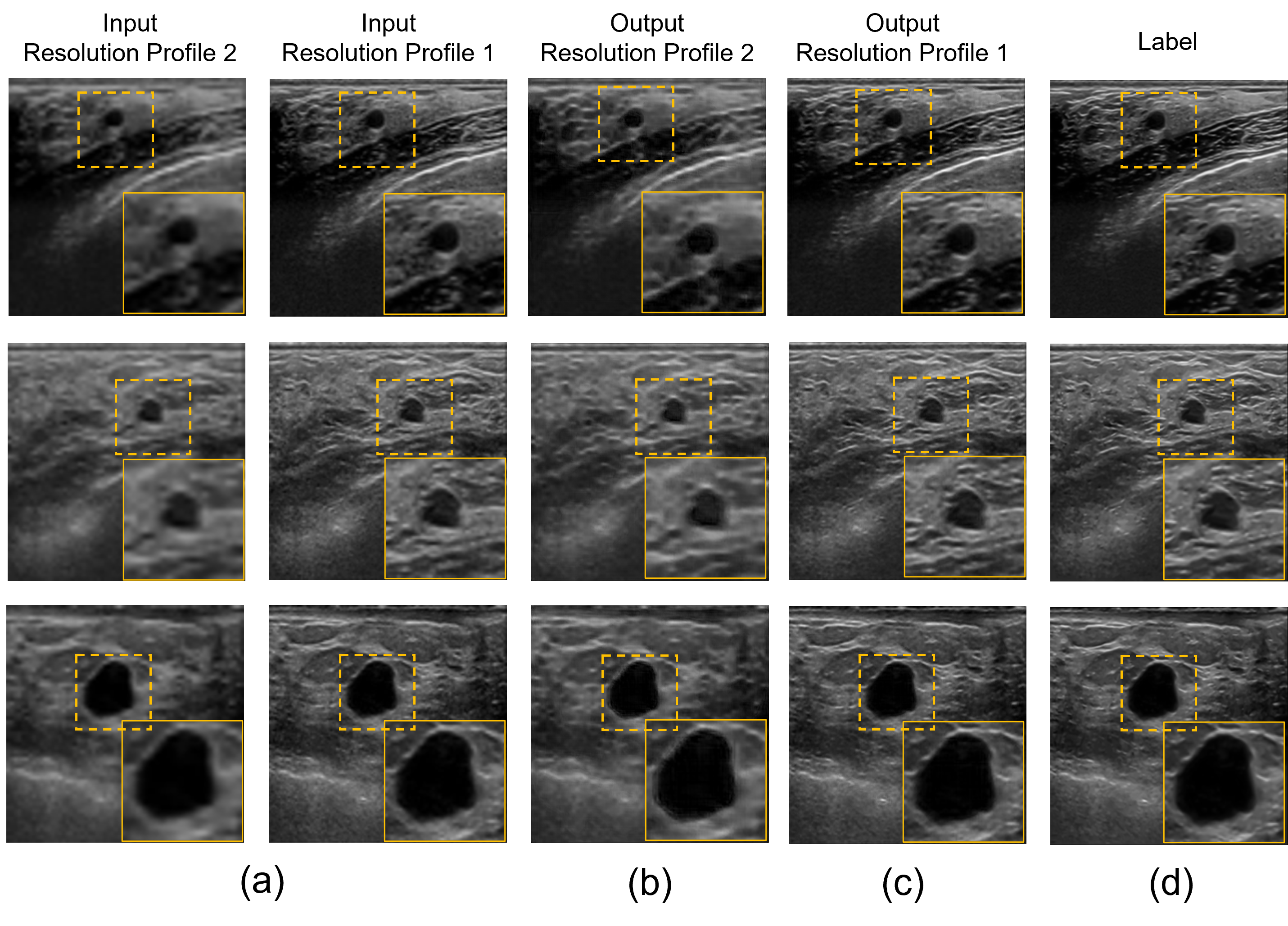

Supplement: Supplementary file 10 [file Image10.PNG]
